# Gendered domestic labor and family leadership: associations with work–family difficulty and expectations for daughters

**DOI:** 10.3389/fsoc.2026.1943089

**Published:** 2026-09-16

**Authors:** Aknur Imankul, Bayan Smagambet

**Affiliations:** Department of sociology, Graduate School of Journalism and Social Sciences, L.N. Gumilyov Eurasian National University, Astana, Kazakhstan

**Keywords:** expectations for daughters, family leadership, gender inequality, Kazakhstan, unpaid domestic labor, work–family difficulty

## Abstract

This article examines whether a female-concentrated division of unpaid domestic labor is associated with a reported household financial-management arrangement, normative endorsement of husband/breadwinner headship, work–family difficulty, perceived barriers, and expectations for daughters in contemporary Kazakhstan. We analyze data from a 2025 national quota survey covering all 17 regions of Kazakhstan and its three cities of republican status (*N* = 2,400; 1,638 married or cohabiting respondents) using binary logistic regression on outcome-specific complete cases (*N* = 1,460–1,523). A one-standard-deviation increase in the domestic-labor index was associated with higher odds that the husband managed the household budget (OR = 1.92), endorsement of the husband or breadwinner as head of household (OR = 1.40), difficulty reconciling work and family (OR = 1.26), and marriage-oriented expectations for daughters (OR = 1.21). The association with naming domestic labor as a barrier to women's participation was not statistically significant after adjustment. The leadership association was stronger among men. Because respondents were individuals, the outcomes capture reported arrangements and attitudes rather than couple-level behavior, and the cross-sectional design precludes causal inference. The results are robust to excluding “difficult to answer” responses rather than coding them as the reference category.

## Introduction

Understanding how families and households organize the everyday work of social reproduction is a central concern for the study of families, households, and relationships. Gender inequality within the household remains one of the most theoretically contested issues in this field. Despite substantial advances in women's educational attainment and labor-force participation across diverse national contexts, women continue to perform a disproportionate share of unpaid domestic labor, even in dual-earner households (Aassve et al., 2014; Bianchi et al., 2000; Coltrane, 2000; Greenstein, 2000; Hochschild and Machung, 1989). This persistent asymmetry suggests that progress toward gender equality in the public sphere has not been matched by comparable transformations within the private domain of family life, a pattern characterized as an uneven and stalled gender revolution (England, 2010).

A growing body of scholarship demonstrates that domestic labor is not merely a practical distribution of tasks but a socially embedded practice through which gender relations, power structures, and normative expectations within families are continuously organized and reproduced (Bourdieu, 2002; Brines, 1994; West and Zimmerman, 1987). Within this tradition, everyday household practices are theorized as mechanisms linking micro-level interactions between family members to macro-level systems of gender stratification. However, the extent to which these dynamics operate across different institutional and cultural contexts remains insufficiently understood, and cross-national research shows that the division of domestic labor varies considerably across institutional and normative settings (Davis and Greenstein, 2004; Knudsen and Wærness, 2008).

Existing research on the division of domestic labor and household authority has largely focused on Western Europe, North America, and East Asia, while post-Soviet and Central Asian societies remain underrepresented in the comparative literature. This geographic imbalance limits the capacity of family studies to access how domestic labor, reported authority arrangements, and gender norms interact under different sociocultural and institutional conditions, and it constrains the field's claim to offer a genuinely international account of family life.

The Republic of Kazakhstan offers a particularly instructive case for the study of families and households. Women account for approximately 48% of the employed population, with female labor-force participation reaching 63%−66% (World Bank, 2024). Women constitute approximately 51%−53% of tertiary-level enrolments (Bureau of National Statistics of the Republic of Kazakhstan, 2024). Yet time-use data indicate that Kazakhstani women allocate substantially more time to unpaid domestic and care work than men (UN Women, 2023), illustrating a sharp disjuncture between formal public equality and persistent domestic inequality within families, a configuration described as a gender paradox (Durrani et al., 2022). While existing Kazakhstani research has examined gender attitudes, fertility, and labor-market outcomes (Ashirbayeva and Smagambet, 2024; Kan, 2023), little attention has been paid to whether everyday domestic arrangements are associated with the distribution of power within families independently of socioeconomic resources and declared gender attitudes.

This article addresses this gap by examining how the reported division of unpaid household labor is associated with two reported family arrangements relevant to household authority, with work–family difficulty is recognition of domestic labor as a barrier, and with expectations for daughters in Kazakhstan. The study makes three contributions. First, it tests whether routine domestic arrangements remain associated with husband-managed budgeting and husband/breadwinner headship after socioeconomic resources and gender attitudes are taken into account. Second, it distinguishes personally experienced work–family difficulty from the explicit recognition of unequal domestic labor as a social barrier. Third, it examines whether domestic arrangements covary with marriage-oriented expectations for daughters without treating a cross-sectional association as evidence of intergenerational transmission. By situating these questions in a post-Soviet Central Asian context, the article broadens the comparative evidence base of family studies. Because the data are across-sectional and reported by individual respondents, all analyses are framed in associational terms.

The article proceeds as follows. The next section reviews the theoretical and empirical literature on domestic labor, household authority, work–family difficulty, and expectations for daughters. The conceptual framework and hypothesis are then presented, followed by the data, measures, and analytical strategy. The results section reports descriptive, bivariate, and multivariate findings together with robustness checks. The article concludes with theoretical implications, limitations, and directions for future research.

## Theoretical background and literature review

Contemporary family scholarship increasingly conceptualizes gender inequality as a relational and structural process embedded in everyday household practices. The gendered division of unpaid domestic labor has emerged as one of the primary sites through which inequality is thought to be reproduced within intimate relationships and across generations. Existing scholarship consistently demonstrates that women continue to perform a disproportionate share of routine housework, caregiving, and emotional labor, even within dual-earner households and formally egalitarian relationships (Aassve et al., 2014; Bianchi et al., 2012; Brines, 1994; Coltrane, 2000; Greenstein, 2000; Lachance-Grzela and Bouchard, 2010).

## Theoretical frameworks

Hochschild and Machung (1989) introduced the concept of the “second shift,” arguing that women's increasing participation in paid employment did not eliminate their responsibility for unpaid domestic labor. West and Zimmerman's (1987) “doing gender” framework offers a complementary lens, conceptualizing gender not as a fixed identity but as something continuously produced through everyday interactions and practices. Domestic labor, from this perspective, functions not only as practical work but as a symbolic process through which femininity, masculinity, and legitimate family roles are enacted and reproduced within daily life.

Bourdieu's (2002) concept of symbolic domination provides a further framework for understanding how domestic inequality becomes normalized through cultural expectations and internalized social norms. Gendered divisions of labor may persist not only because of material inequalities but because caregiving, emotional support, and household management are socially constructed as feminine responsibilities, rendering domestic inequality simultaneously invisible and morally legitimized. Symbolic domination is theorized to operate not through overt coercion but through habituated dispositions that make unequal arrangements appear natural.

Economic explanations retain substantial influence. Relative resources theory (Blood and Wolfe, 1960) posits that partners with greater economic resources possess stronger bargaining power and are better positioned to avoid undesirable domestic responsibilities. (Brines (1994) demonstrated that women's economic dependency contributes to reproducing traditional divisions of labor even in dual-earner households. Extending this exchange-bargaining logic, Bittman et al. (2003) found that women reduce their housework as their relative earnings rise, but only up to the point of equal contribution, beyond which additional income does not further reduce it, so that gender rather than money governs the division. Time-availability theory (Coverman, 1985) similarly argues that domestic labor reflects differences in paid employment participation and available time. A direct test of the relative-resources mechanism, however, requires information on partners' resources in relation to one another—the partner's earnings, or each partner's share of household income—rather than the absolute resources of a single respondent. However, empirical studies repeatedly find that even where women participate equally in paid employment, they continue to perform disproportionate shares of unpaid labor, a finding that points toward the structural and normative dimensions of domestic inequality.

## Domestic labor, reported authority, and work–family constraints

Recent scholarship has moved beyond documenting unequal task distributions toward examining how domestic practices relate to symbolic power, relational authority, and emotional regulation. Carlson et al. (2020) show that inequalities in household labor are closely associated with perceptions of fairness, communication, and relationship satisfaction. An important conceptual development concerns “invisible” domestic labor: the cognitive and emotional work of planning, coordinating, and anticipating needs (Daminger, 2019). Aviv et al. (2025) argue that cognitive labor is psychologically demanding and associated with diminished wellbeing among women (see also Ciciolla and Luthar, 2019). Research in the Journal of Family Studies further shows that women may assume disproportionate responsibility for information-seeking and the administration of parental benefits (Flinkfeldt and Höglund, 2024), while Daminger et al. (2025) demonstrate that household inequalities are reproduced through divergent expectations of gratitude and recognition. We draw on this literature to motivate the study, while noting that our survey measures visible task allocation rather than cognitive, emotional, or administrative labor.

Barnwell (2024) extends the concept of invisible family labor into the domain of what she terms “not doing family,” showing that even severed family relationships require continuous emotional management and normative negotiation. Drawing on Morgan's (2011) concept of family practices and Finch's (2007) notion of family display, this work suggests that normative expectations regarding family roles generate forms of responsibility that are managed largely off-display and are therefore underestimated by research attending only to observable task distributions. Unequal domestic labor also translates into structural constraints on women's labor-market participation (Samtleben and Müller, 2022), and institutional reforms such as flexible work arrangements do not automatically produce more egalitarian practices, their effects being mediated by prevailing gender ideologies (Cao and Wang, 2025). The transition to parenthood is a critical juncture that frequently reinforces traditional divisions of care even among egalitarian couples (Alsarve and Glatz, 2025; Craig and Mullan, 2010; Estlein et al., 2024).

## Intergenerational expectations and the post-Soviet context

Domestic labor inequalities may also be reflected in expectations directed toward the next generation. Research on the “hidden curriculum” of the family (Nitsche and Grunow, 2016) suggests that gender roles are learned partly through observation of routine household practices and parental modeling. Studies of fatherhood show that men may conceptualize their domestic participation through comparative frameworks that obscure persistent inequalities in mental load (Squires et al., 2025). Contemporary research also connects gendered household arrangements to broader demographic change: perceived gender conflict and unequal expectations are associated with weaker support for marriage and childbearing in East Asia (Lee et al., 2025; Taniguchi and Kaufman, 2014). The present study does not observe transmission across generations; it tests the narrower proposition that current domestic arrangements are associated with the expectations respondents report for daughters.

Despite the expansion of international scholarship, Central Asian and post-Soviet societies remain comparatively underrepresented in family studies. This is a significant gap because post-Soviet societies combine high levels of women's labor-force participation, a legacy of Soviet-era gender policy, with persistent cultural expectations regarding caregiving. In Central Asia specifically, post-Soviet nation-building has been accompanied by a revalorization of traditional gender roles and the family (Cleuziou and Direnberger, 2016; Kudaibergenova, 2018), and women may pursue security and status through conformity to prescribed family roles, a dynamic Kandiyoti (1988) termed the patriarchal bargain. Kan (2023) demonstrates that greater equality in domestic labor is associated with more egalitarian attitudes but also lower short-term fertility intentions in Kazakhstan, and Ashirbayeva and Smagambet (2024) show that motherhood remains strongly institutionalized, with deviations subject to stigma. These findings indicate that domestic labor in Kazakhstan is embedded in broader systems of symbolic legitimacy and reproductive expectation.

## Conceptual framework and hypotheses

Drawing on the doing-gender perspective (West and Zimmerman, 1987) and Bourdieu's (2002) framework of symbolic domination, we expect more female-concentrated domestic arrangements to be associated with more traditional reported family arrangements—both in how household finances are reported to be managed and in normative views about who should head the family. Relative-resources and bargaining accounts (Blood and Wolfe, 1960; Brines, 1994) inform this expectation, but the survey measures only the respondent's own income and contains no measure of the partner's resources or of relative economic contributions within the couple. The hypotheses are therefore not framed as tests of a bargaining mechanism; the analysis can establish only whether the observed patterns are compatible with one. The hypotheses concern associations, consistent with the cross-sectional design.

**H1:** A more traditional (female-concentrated) division of unpaid domestic labor is positively associated with the likelihood that the husband manages the household budget.

**H2:** A more traditional division of domestic labor is positively associated with endorsement of the husband or breadwinner as head of household.

**H3:** A more traditional division of domestic labor is positively associated with experiencing work–family difficulty (H3a) and with naming unequal domestic labor as a barrier to women's participation (H3b).

**H4:** A more traditional division of domestic labor is positively associated with marriage-oriented rather than career-oriented expectations for daughters.

Domestic labor, reported financial-management arrangements, normative endorsement of family leadership, gender attitudes, work–family difficulty, and expectations for daughters are conceptualized as related dimensions of a broader system of gender inequality. Financial management and family leadership are kept analytically separate: the first records a reported arrangement, the second a normative endorsement, and neither is treated as a direct measure of decision-making power within the couple. The study tests the association of domestic-labor asymmetry with each outcome net of resources and attitudes; it does not establish causal ordering or couple-level agreement.

## Methods

### Data and sample

This study draws on secondary data from the national quota survey “Gender Stereotypes in Various Spheres of Kazakhstani Society,” conducted in 2025 by the Kazakhstan Institute for Public Development. Kazakhstan is a middle-income post-Soviet state in Central Asia, characterized by Soviet institutional legacies, a predominantly Muslim cultural heritage, and rapid social transformation since independence in 1991. The original sample comprised 2,400 respondents aged 18 years and older, distributed across all 17 regions of Kazakhstan and the three cities of republican status (Astana, Almaty, and Shymkent), and covering both urban and rural settlements. Sample sizes per region were set proportionally to regional population figures from the Bureau of National Statistics, ranging from 27 respondents in the smallest region to 177 in the largest, with a corresponding split of urban and rural allocations and of Kazakh- and Russian-language questionnaires in each region. Within each sampled settlement, households were selected by route/random walk and individual respondents within households were selected by quota on gender and age, matched to population parameters for the corresponding region and settlement type; interviewers continued sampling until regional quotas were filled. Because this is a quota rather than a probability design, a conventional response rate was not computed and no additional post-stratification weights were applied beyond the quota targets themselves; the sample is therefore consistent with the characteristics of the adult population of Kazakhstan by region, settlement type, gender, and age based on quotas, rather than representative of it in the probability-sampling sense, and we do not extend this claim beyond Kazakhstan itself. The analytic sample was restricted to married or cohabiting respondents because the focal questions concern current intra-household arrangements, yielding 1,638 respondents (855 women and 783 men). Of these, 1,528 had complete data on all covariates. “Difficult to answer” responses were then excluded separately for each dependent variable, producing outcome-specific model samples of 1,460–1,523.

### Measures

#### Division of domestic labor

The key independent variable was constructed from four survey items asking who in the household usually performs cooking, cleaning, dishwashing, and childcare. For each task, respondents chose one of five original response options: husband, wife, jointly, another household member, or “no such duty in the family” (the last option applying, for example, to childcare when the household has no children), plus a “difficult to answer” option. Each item was coded +1 where the task is performed mainly by the woman, −1 where it is performed mainly by the man, and 0 where the “jointly,” “another household member,” or “no such duty” options were selected. “Difficult to answer” responses on these items, which affected 16 respondents (1.0%) on at least one task, were also coded 0 so that those respondents were retained in the index; a sensitivity analysis treating them as missing is reported below. We acknowledge that collapsing “jointly” and “another household member” into a single midpoint conflates two substantively different arrangements, which may matter particularly in extended households where parents or other relatives share caregiving; the “no such duty” option, used mainly for childcare among childless respondents, is likewise absorbed into the same midpoint rather than treated as structurally missing. In the analytic sample, 181 married or cohabiting respondents (11.1%) reported that at least one task was performed by another household member, confirming that this is a non-trivial subgroup rather than a coding artifact. We therefore re-estimated a revised index in which “another household member” and “no such duty” responses are treated as missing on the affected item, rather than equivalent to joint performance, retaining respondents with valid data on at least three of the four items and rescaling to the same −4 to +4 metric (Cronbach's α = 0.77; mean inter-item *r* = 0.47; correlation with the original four-category index *r* = 0.97). The revised-index results are reported alongside the other robustness checks below and are consistent with the main analysis. The four items were summed to form an index ranging from −4 (fully male-concentrated) to +4 (fully female-concentrated). Internal consistency is acceptable (Cronbach's α = 0.76; mean inter-item *r* = 0.45); the three routine chores cohere strongly, whereas childcare loads more weakly, and a sensitivity analysis excluding childcare is reported below. The index captures the visible allocation of household tasks and does not measure cognitive or emotional labor.

#### Reported family arrangements and other outcomes

Two binary indicators were used, and they measure conceptually distinct things. The first, husband-managed household budget, is a reported financial-management arrangement rather than an indicator of household authority: it records who is said to administer the budget, not who decides how money is spent, and administering a budget may in some households be a routine administrative responsibility rather than an exercise of power. It derives from the same household-tasks item used to build the domestic-labor index (“Who manages the household budget?,” with response options husband, wife, jointly, another household member, or no such duty), recoded 1 = husband, 0 = jointly, wife, another household member, or no such duty. The second, endorsement of the husband or breadwinner as head of household, derives from a separate item (“Who do you think should be the head of the family?,” with response options husband, wife, whoever provides for the family, equal partners, or difficult to answer), recoded 1 = husband or whoever provides for the family, 0 = wife or equal partners. Budget management is therefore treated throughout as a reported financial-management arrangement whose relationship to household authority is an open question rather than an assumption, and the leadership item deliberately combines two distinct response options (husband; provider) because both were volunteered as alternatives to equal partnership in the original instrument; we report the two source options separately in the Discussion of construct boundaries below. Work–family outcomes were (1) difficulty reconciling paid work and family (1 = rather or entirely unable to reconcile) and (2) naming the unequal distribution of domestic duties as a barrier to women's participation. Expectations for daughters were measured by a single item on what is more important for a daughter, recoded as marriage-oriented (1) vs. career-oriented (0). For every dependent variable, “difficult to answer” was treated as missing and excluded from that model rather than coded 0. These single-item outcomes capture reported arrangements and normative endorsements rather than independently observed couple-level behavior.

#### Controls

Models adjust for respondent gender (female), age (years), education (ordinal), personal income (ordinal band), settlement type (urban vs. rural), the presence of children in the household, and traditional gender attitudes (agreement that the primary role of women is to be homemakers, 1–4). Two measurement limitations are noted: the children item records the number of minor children (none, one, two, three, four, or more) and is used here as a binary indicator of whether minor children are present, so it does not distinguish ages within that group; and the income item reflects personal rather than household income.

## Analytical strategy

Analysis proceeded in three stages. First, descriptive statistics were computed by respondent gender (Table 1) using valid item-specific denominators. Second, Spearman correlations examined bivariate associations with pairwise exclusion of outcome-specific nonresponse (Figure 1). Third, binary logistic regression models estimated the association of domestic-labor asymmetry with each outcome, adjusting for the full set of covariates (Figure 2; full estimates are reported in Supplementary Tables S1–S5). Models were estimated on respondents complete on all covariates and valid on the focal outcome, so *N* varies from 1,460 to 1,523. We report odds ratios with 95% confidence intervals, model *N*, McFadden's *R*^2^, and likelihood-ratio tests. We additionally report the odds ratio per standard deviation of the index and its average marginal effect. Robustness checks comprised a categorical index, a three-task index excluding childcare, gender-stratified models, a gender × index interaction, and a sensitivity analysis coding “difficult to answer” responses on the index items as 0. Analyses were performed in Python 3.12 (Python Software Foundation, Wilmington, DE, USA) using maximum-likelihood estimation; tests were two-tailed and estimates are associational.

**Table 1:** Descriptive statistics of key variables by respondent gender.

Variable	Women (*n* = 855)	Men (*n* = 783)
Domestic labor index (−4 to +4), *M* (SD)	2.50 (1.47)	2.58 (1.38)
Time on housework (0–6), *M* (SD)	3.55 (1.40)	1.92 (1.68)
Husband-managed household budget (%)	18.4	27.2
Husband/breadwinner headship (%)	55.1	74.3
Work–family difficulty (%)	10.8	8.0
Domestic labor named as a barrier (%)	32.2	31.0
Traditional gender attitudes (1–4), *M* (SD)	2.84 (1.01)	3.23 (0.91)
Marriage-oriented expectation for daughters (%)	47.8	49.5

Percentages use valid item-specific denominators after excluding “difficult to answer” responses (women n = 809–851; men n = 745–782). The sample is restricted to married or cohabiting respondents (N = 1,638; 855 women and 783 men).

**Figure 1:**
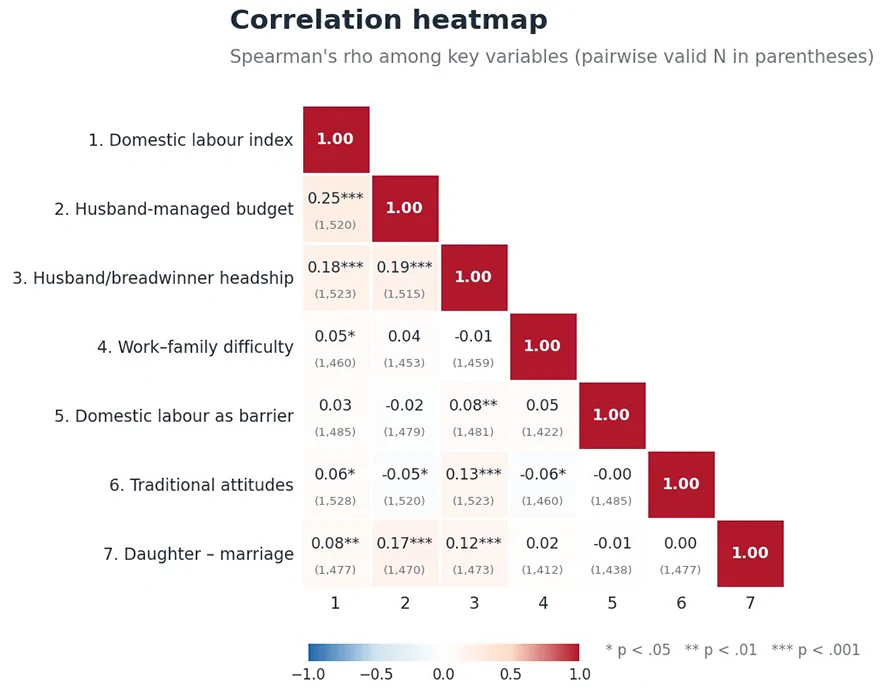
Spearman correlations among key variables. Spearman's rho with pairwise valid responses (*N* = 1,412–1,528); respondents are complete on model covariates. Cell labels report rho and pairwise *N*; *p < 0.05, **p < 0.01, and ***p < 0.001.

**Figure 2:**
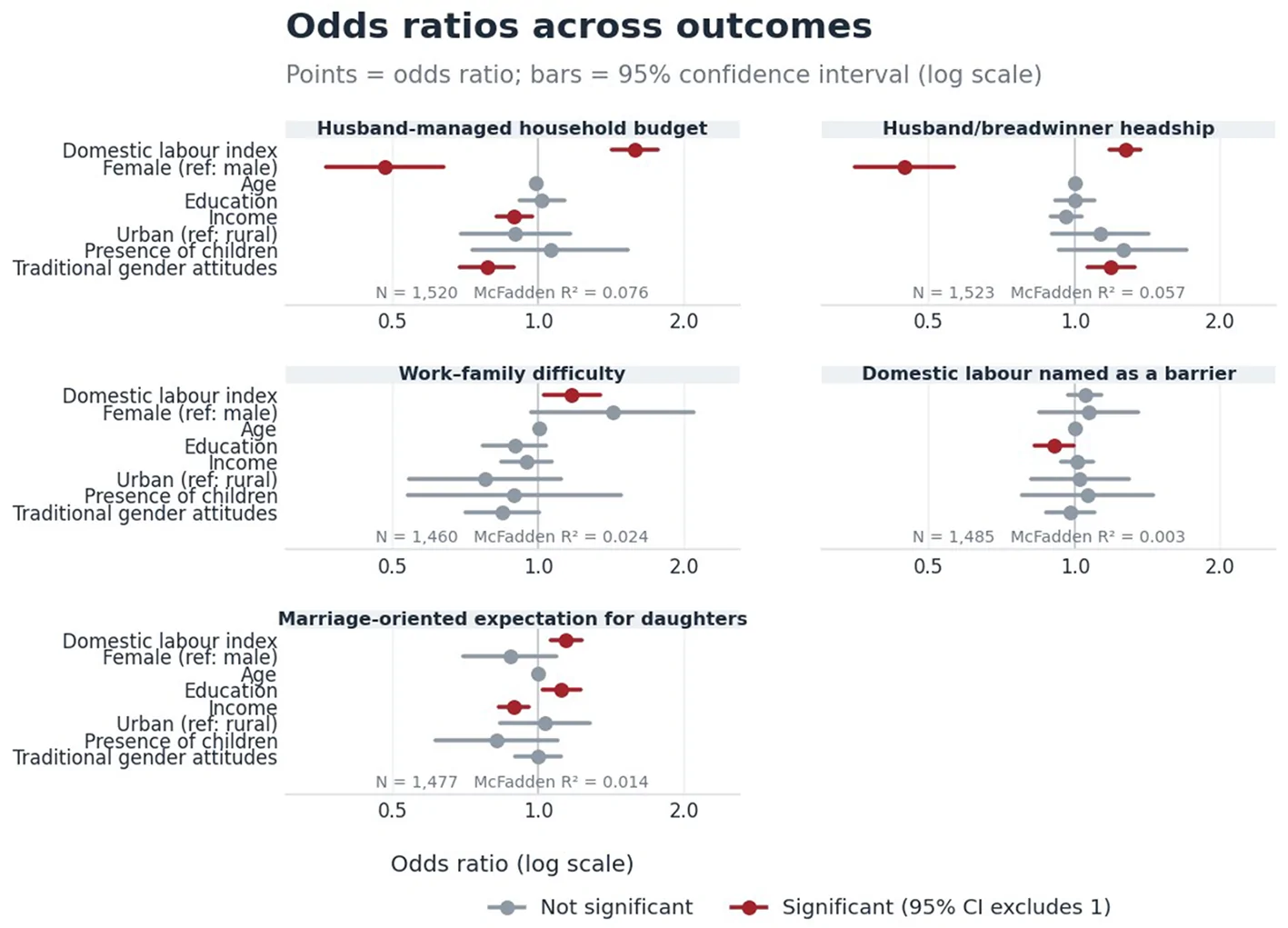
Adjusted odds ratios across five binary outcomes. Outcome-specific complete cases: *N* = 1,460–1,523. Points are odds ratios and bars are 95% confidence intervals on a logarithmic scale. Red markers indicate intervals that exclude 1. Full numerical estimates and model statistics appear in Supplementary Tables S1–S5.

## Ethics

This project was conducted with institutional ethics clearance from the Digital Resource Centre for Research Ethics of L.N. Gumilyov Eurasian National University, Astana, Kazakhstan (Expert Opinion No. 9, 14 March 2025). All participants provided written informed consent; data were fully anonymized before transfer to the research team under a formal data-use agreement. The studies were conducted in accordance with the local legislation and institutional requirements. Written informed consent for participation was not required from the participants or the participants' legal guardians/next of kin in accordance with the national legislation and institutional requirements. Participants were 2,400 individuals aged 18 years and older. Households were used solely as sampling units: one adult respondent was selected within each household according to gender and age quotas. Families were not enrolled or studied as collective participants, no household member other than the selected respondent was interviewed, and no minors participated in or were the subject of the research. The study involved no non-governmental organizations, service providers, social workers or other professional intermediaries, and no participants were recruited through clinical, welfare or social-service settings. Before each interview, respondents received written information about the purpose of the study, the voluntary nature of participation, the intended use of the data and the confidentiality safeguards, and provided written informed consent; respondents could decline any individual question or discontinue participation at any point without consequence. The questionnaire collected no names or other direct identifiers, and questionnaire numbers and settlement details were used only for fieldwork quality control. The ethics opinion identified items concerning discrimination, sexual harassment, income, ethnicity, religion and family status as potentially sensitive, and required that interviews be administered neutrally and in private and that interviewers be briefed on handling sensitive topics and maintaining confidentiality; on that basis the study was classified as minimal risk. The analytical dataset was fully de-identified before transfer to the research team under a formal data-use agreement, and results are reported only in aggregate form.

## Results

### Descriptive statistics

Descriptive statistics reveal a clear gender imbalance in the distribution of unpaid domestic labor (Table 1). Women report substantially more time on routine household tasks (*M* = 3.55 on the 0-6 scale) than men (*M* = 1.92). The task-allocation index is strongly female-concentrated and similar whether reported by women (*M* = 2.50) or men (*M* = 2.58). Using valid outcome-specific denominators, men more often than women report husband/breadwinner headship (74.3% vs. 55.1%) and husband-managed budgeting (27.2% vs. 18.4%).

### Bivariate associations

Recomputed correlations are modest and consistent with the multivariate results (Figure 1). The index is most strongly associated with husband-managed budgeting (*r*_*s*_ = 0.25) and husband/breadwinner headship (*r*_*s*_ = 0.18), and more weakly with marriage-oriented expectations for daughters (*r*_*s*_ = 0.08) and traditional attitudes (*r*_*s*_ = 0.06). Its association with work–family difficulty is small (*r*_*s*_ = 0.05), while the association with naming domestic labor as a barrier is near zero (*r*_*s*_ = 0.03). Pairwise valid *N* ranges from 1,412 to 1,528. These small coefficients indicate that statistically robust multivariate associations should be interpreted as modest.

### Reported family arrangements

The logistic models support H1 and H2 (Figure 2; Supplementary Tables S1, S2). A more female-concentrated division of domestic labor is associated with higher odds that the husband manages the household budget [OR per index point = 1.58, 95% CI (1.42, 1.77), and *p* < 0.001; *N* = 1,520; per standard deviation, OR = 1.92, 95% CI (1.64, 2.24)]; the average marginal effect is +7.4 percentage points per index point. A similar pattern holds for husband/breadwinner headship [OR = 1.27, 95% CI (1.18, 1.37), and *p* < 0.001; *N* = 1,523; per standard deviation, OR = 1.40, 95% CI (1.26, 1.57); average marginal effect = +5.1 percentage points]. Model fit is modest (McFadden *R*^2^ = 0.076 and 0.057, respectively).

Because outcomes are reported by individual respondents rather than measured at the couple level, we tested whether the index associations differ by respondent gender. For husband/breadwinner headship, the gender × index interaction is statistically significant [interaction OR = 0.74, 95% CI (0.63, 0.86), and *p* < 0.001]: the association is stronger among men [OR = 1.52, 95% CI (1.34, 1.71)] than among women [OR = 1.12, 95% CI (1.01, 1.23)]. For husband-managed budgeting, the interaction does not reach the 0.05 threshold [interaction OR = 0.81, 95% CI (0.65, 1.01), and *p* = 0.066], while the index association is present among women [OR = 1.44, 95% CI (1.23, 1.68)] and men [OR = 1.73, 95% CI (1.48, 2.03)].

### Work–family difficulty and perceived barriers

Support for H3 is partial (Figure 2; Supplementary Tables S3, S4). A more female-concentrated division of domestic labor is associated with higher odds of work–family difficulty [OR = 1.17, 95% CI (1.02, 1.34), and *p* = 0.020; *N* = 1,460; per standard deviation, OR = 1.26, 95% CI (1.04, 1.52)], although the average marginal effect is small (+1.4 percentage points) and model fit is low (McFadden *R*^2^ = 0.024). By contrast, the association with naming domestic labor as a barrier is not statistically significant after adjustment [OR = 1.05, 95% CI (0.97, 1.14), and *p* = 0.224; *N* = 1,485; McFadden *R*^2^ = 0.003]. H3 is supported for experienced work–family difficulty (H3a), but not for explicit recognition of domestic labor as a barrier (H3b).

### Intergenerational expectations

Results support H4 (Figure 2; Supplementary Table S5). A more female-concentrated division of domestic labor is associated with higher odds of marriage-oriented rather than career-oriented expectations for daughters [OR = 1.14, 95% CI (1.06, 1.23), and *p* < 0.001; *N* = 1,477; per standard deviation, OR = 1.21, 95% CI (1.08, 1.34); average marginal effect = +3.2 percentage points]. Traditional gender attitudes are not statistically significant in this model. The single-item outcome is a reported expectation, not evidence that intergenerational transmission has occurred.

### Robustness

Coding “difficult to answer” responses on the index items as 0, rather than excluding them, does not alter the substantive conclusions: sensitivity models excluding these responses instead yield the same support pattern across H1–H4. A categorical specification shows graded associations: compared with an index ≤ 0, the most female-concentrated group (index ≥ 3) has higher odds of husband-managed budgeting [OR = 3.08, 95% CI (2.00, 4.75)], leadership endorsement [OR = 2.17, 95% CI (1.61, 2.92)], work–family difficulty [OR = 2.13, 95% CI (1.14, 3.98)], and marriage-oriented expectations [OR = 1.57, 95% CI (1.17, 2.10)], but not barrier recognition. A three-task index excluding childcare retains the budget, leadership, and daughter-expectation associations; the work–family estimate remains positive but less precise. Gender-stratified and interaction models reproduce the stronger leadership association among men. A further robustness check re-estimates all five models using the revised index that treats “another household member” and “no such duty” as missing rather than as equivalent to joint performance (see Section Measures). Effect sizes shift modestly but the overall pattern of support for H1–H4 is unchanged: husband-managed budgeting [OR per SD = 1.74, 95% CI (1.48, 2.05), and *N* = 1,405], leadership endorsement [OR per SD = 1.39, 95% CI (1.24, 1.56), and *N* = 1,404], work–family difficulty [OR per SD = 1.38, 95% CI (1.11, 1.72), and *N* = 1,350]—somewhat stronger than in the main specification—barrier recognition remains non-significant [OR per SD = 1.06, 95% CI (0.94, 1.19), and *N* = 1,369], and marriage-oriented expectations for daughters [OR per SD = 1.21, 95% CI (1.09, 1.35), and *N* = 1,361]. This indicates that the substantive conclusions do not depend on collapsing shared and other-member task performance into a single category. Treating “difficult to answer” responses on the index items as missing rather than as 0 (*N* = 1,622) likewise leaves the estimates essentially unchanged—husband-managed budgeting OR = 1.59, 95% CI (1.42, 1.78); husband/breadwinner headship OR = 1.27, 95% CI (1.18, 1.38); work–family difficulty OR = 1.19, 95% CI (1.04, 1.37); barrier recognition OR = 1.06, 95% CI (0.98, 1.15); expectations for daughters OR = 1.13, 95% CI (1.05, 1.22)—confirming that this coding choice, which affected 1.0% of the analytic sample, is immaterial to the findings. A final check addresses the construction of the husband/breadwinner-headship indicator itself. Estimating the “husband” and “breadwinner” response options as separate binary outcomes (each against the remaining response options combined) shows that the household-authority association is carried specifically by the husband response [OR per standard deviation = 1.36, 95% CI (1.24, 1.50), and *p* < 0.001] and not by the breadwinner response considered alone [OR per standard deviation = 1.08, 95% CI (0.87, 1.34), and *p* = 0.466]; excluding the small number of respondents who named the wife as head (1.8% of valid responses) from the reference category, rather than retaining them alongside egalitarian responses, leaves the main composite association essentially unchanged [OR per standard deviation = 1.43, 95% CI (1.28, 1.59), vs. 1.40 in the main specification]. These checks are discussed further in the Section Limitations.

## Discussion

The findings provide consistent evidence that the reported gendered division of unpaid domestic labor is associated with several dimensions of family inequality after socioeconomic resources and declared gender attitudes are taken into account. Associations are strongest for the reported budget arrangement and the leadership endorsement, weaker but present for work–family difficulty and expectations for daughters, and absent for explicitly naming domestic labor as a barrier. The cross-sectional, individual-level design means that these results are consistent with, but do not demonstrate, the theoretical mechanisms outlined earlier.

The pattern aligns with the doing-gender framework (West and Zimmerman, 1987) and Bourdieu's (2002) account of symbolic domination, in which inequality is sustained through routine practices that acquire the appearance of normality. It is also compatible with bargaining and relative-resources perspectives (Blood and Wolfe, 1960; Brines, 1994), but they cannot test them. Relative-resources theory concerns partners' resources in relation to one another, whereas the survey records only the respondent's personal income and includes no measure of the partner's earnings or of each partner's share of household income. Compatibility with this perspective is therefore the strongest claim the present data support, and the results should not be read as evidence that a bargaining process is operating. The persistence of the associations after adjustment for income, education, and attitudes suggests that domestic arrangements, reported budget management, and endorsement of husband/breadwinner headship are related beyond these measured resource differentials. The McFadden pseudo-*R*^2^ values reported here (0.003–0.076) are low in absolute terms but are consistent with what is typically observed for individual-level logistic regression models of attitudinal and behavioral outcomes in the social sciences, where a single predictor rarely accounts for a large share of deviance even when it is a statistically robust and theoretically central one; McFadden values of 0.02–0.20 are commonly treated as acceptable in this literature, so the low pseudo-*R*^2^ here should be read as an indication that many factors beyond domestic-labor allocation shape these outcomes, not as evidence against the association itself. At the same time, statistical significance and practical significance are not equivalent. The average marginal effects translate the odds ratios into percentage-point terms that are more directly interpretable: a one-point increase in the domestic-labor index is associated with a 7.4 percentage-point increase in the probability of husband-managed budgeting and a 5.1 percentage-point increase in endorsing husband/breadwinner headship, whereas the corresponding shifts for work–family difficulty (+1.4 points) and daughter expectations (+3.2 points) are modest in absolute size even though they are statistically robust. We treat the budget and leadership associations as both statistically and practically meaningful, and the work–family and daughter-expectation associations as statistically robust but practically modest.

Construct boundaries are central to interpretation. Managing the household budget is not synonymous with controlling financial decisions and may itself constitute administrative labor: the person who administers the budget may be executing decisions taken jointly or by someone else, so the arrangement can reflect a division of routine responsibility as much as a distribution of power. The association reported here shows that a female-concentrated division of domestic work covaries with husband-managed budgeting; whether that arrangement confers, reflects, or is independent of financial authority cannot be determined from these data, and the two should not be treated as equivalent. Likewise, the leadership indicator combines endorsement of the husband as head with endorsement of the provider as head, so it captures husband/breadwinner headship rather than exclusively male authority. These measures are therefore treated as limited indicators of reported family arrangements and normative endorsements, not as measures of household authority as such. The stronger association between domestic labor and leadership among male respondents further indicates that the results partly reflect gendered perceptions and reports rather than independently verified couple-level behavior.

The partial support for H3 is substantively informative. Respondents may experience difficulty reconciling work and family without explicitly identifying unequal domestic labor as a structural barrier to women's participation. This disjunction is consistent with normalization: a routine arrangement can generate strain while remaining weakly recognized as a social mechanism. At the same time, the near-zero explanatory power of the barrier model cautions against attributing the non-significant result solely to normalization; the single-item measure may also lack sensitivity.

The association between domestic labor and marriage-oriented expectations for daughters is compatible with the hidden-curriculum thesis (Nitsche and Grunow, 2016), but it does not establish that household practices cause or transmit those expectations. The finding is better read as evidence that present domestic arrangements and reported expectations cluster together, motivating longitudinal and multi-informant research on intergenerational processes.

In Kazakhstan, high women's educational attainment and labor-force participation coexist with a strongly female-concentrated division of routine domestic work. The observed associations extend post-Soviet gender-contract research by showing how visible task allocation covaries with reported financial-management arrangements, normative endorsement of husband/breadwinner headship, and expectations for daughters in Central Asia. They also complement recent Journal of Family Studies research on women's administrative responsibility for parental benefits (Flinkfeldt and Höglund, 2024) and on gendered cognitive labor among fathers (Squires et al., 2025). Because the survey does not capture cognitive, emotional, or administrative labor, the present estimates likely represent only part of the relevant inequality.

## Limitations

Several limitations qualify these conclusions. The cross-sectional design precludes causal inference and cannot rule out reverse association or shared unmeasured causes. Respondents are individuals rather than couples, so the outcomes reflect reports and normative endorsements rather than couple-level agreement, and the analysis cannot establish whether partners would describe their arrangements in the same terms.

Several measurement caveats set out in the Methods bear on how the estimates should be read. The authority, barrier, and daughter-expectation outcomes are single items and may be affected by social-desirability bias. Budget management is a reported financial-management arrangement rather than a measure of financial control or decision-making power, and its relationship to household authority is not established by these data; the husband/breadwinner-headship indicator combines endorsement of the husband as head with endorsement of the breadwinner as head, coded 1, against wife, equal partners, or “does not matter,” coded 0. Because the response “wife” (1.8% of valid answers) is itself a headship response, only female-headed, its inclusion in the reference category means that category mixes non-hierarchical endorsements (equal partners; does not matter) with this small hierarchical-but-female-headed group; a sensitivity check that removes these respondents rather than retaining them in the reference category leaves the association essentially unchanged (OR per standard deviation = 1.43 vs. 1.40 in the main specification). A further check estimating the husband and breadwinner responses as separate binary outcomes shows that the association is carried by the husband response (OR per standard deviation = 1.36, *p* < 0.001) and not by the breadwinner response considered alone (OR per standard deviation = 1.08, *p* = 0.47), so the indicator is best read as capturing endorsement of the husband, specifically, as head of household, with the gender-neutral breadwinner criterion contributing little independently. The domestic-labor index captures visible routine tasks: childcare loads more weakly than the three chores, and cognitive, emotional, and administrative labor are not measured. The index also collapses jointly performed tasks and tasks performed by another household member into a single midpoint category, and childcare items where the respondent reports “no such duty” are absorbed into the same category; a revised index treating the latter two options as missing rather than as equivalent to joint performance yields substantively similar conclusions (see Robustness), but the distinction between egalitarian couples and extended households in which grandparents or other relatives share caregiving is not separately modeled here and would benefit from a dedicated household-composition measure in future waves. Among the controls, the children indicator records whether minor children are present but does not distinguish their ages, and personal income is used instead of household or relative partner income.

The sample also bounds the claims that can be made. It was constructed using a quota design (region, settlement type, gender, and age matched to national population parameters) rather than probability sampling; a conventional response rate was therefore not computed and no post-stratification weights beyond the quota targets were applied, so the sample is described as consistent with the characteristics of the Kazakhstani population based on quotas, rather than as representative of it in the design-based, probability-sampling sense. The achieved sample closely tracked the planned allocation—for example, 174 respondents in Astana against a target of 174 and 177 in Almaty region against a target of 177, with regional totals matching the planned sampling frame across all 17 regions and the three cities of republican status—indicating that the quota was fulfilled as designed, though this does not substitute for a probability-based response rate. Beyond the design, the findings describe the contemporary social context of Kazakhstan specifically, shaped by its particular combination of Soviet-era institutional legacies, rapid post-independence social change, and predominantly Muslim cultural heritage; they should not be assumed to generalize automatically to other Central Asian or post-Soviet countries, which differ from Kazakhstan and from one another in religious composition, economic structure, and the pace and direction of gender-policy reform since 1991. Comparative empirical evidence from neighboring countries would be required before any regional claim could be supported, and systematic cross-national comparison of domestic-labor arrangements, reported household authority, and expectations for daughters across post-Soviet countries is accordingly a promising direction for future research, given the contribution the present study makes to extending the geographical scope of family sociology beyond Western and East Asian settings.

Finally, 110 partnered respondents lack at least one covariate, and outcome-specific nonresponse removes a further 5–68 cases from individual models. Although a sensitivity analysis leaves the conclusions unchanged, future research should use multiple imputation or planned missing-data procedures alongside longitudinal dyadic designs.

## Conclusion

This study asked whether a female-concentrated division of unpaid domestic labor is associated with a reported household financial-management arrangement, normative endorsement of husband/breadwinner headship, work–family difficulty, perceived barriers, and expectations for daughters in Kazakhstan. Domestic-labor asymmetry is associated most clearly with husband-managed budgeting and husband/breadwinner headship, more weakly with work–family difficulty and marriage-oriented expectations for daughters, and not with explicitly naming domestic labor as a barrier. The leadership association is stronger among male respondents, reinforcing the need to interpret these outcomes as individual reports.

The study contributes to family scholarship in three ways. It provides evidence that routine domestic arrangements covary with a reported financial-management arrangement and with normative endorsement of husband/breadwinner headship after measured resources and attitudes are taken into account; it separates experienced work–family difficulty from explicit recognition of domestic inequality as a barrier; and it documents an association between present household arrangements and expectations for daughters without construing that association as observed intergenerational transmission. Evidence from Kazakhstan broadens a literature still dominated by Western and East Asian settings.

The findings motivate policy-relevant research on how workplace conditions, family policy, and the distribution of unpaid labor operate together; the present design cannot establish which intervention would change household arrangements. Longitudinal, dyadic, and mixed-method studies are needed to test the causal processes implied by doing-gender, bargaining, and symbolic-domination perspectives and to capture the invisible labor omitted from the current survey.

## Data Availability

The dataset analyzed in this study is not publicly available. It was provided to the research team under a formal data-use agreement that does not permit open access or public sharing of the individual-level data. Requests to access the data may be directed to the corresponding author, subject to the terms of that agreement.
