# Supplementary material for: Gendered domestic labor and family leadership: associations with work–family difficulty and expectations for daughters
Source: Front Sociol. 2026 Sep 16;11:1943089. doi: 10.3389/fsoc.2026.1943089 (PMC13624119; doi:10.3389/fsoc.2026.1943089)
Supplement: Supplementary file 1 [file Supplementary_File_1.docx]

**SUPPLEMENTARY MATERIAL**

*Supplementary Tables S1–S5 report the full binary logistic regression model for each of the five outcomes, adjusting for the covariates shown. Each cell reports the odds ratio (OR) with its 95% confidence interval and p-value; odds ratios are per one-unit increase in the predictor, and the domestic-labor index is on its natural −4 to +4 scale (per index point). The focal domestic-labor-index row and the model-fit statistics (n, McFadden R²) are entered from the article; bracketed entries ([OR], [LL, UL], [p], [χ²(df)]) are placeholders for the covariate estimates and the likelihood-ratio test, to be filled from the fitted models. Delete this note before submission.*

**Supplementary Table S1 |** Binary logistic regression predicting husband-managed household budget.

| **Predictor** | **OR** | **95% CI** | **p** |
| --- | --- | --- | --- |
| **Domestic-labor index (per index point)** | **1.58** | **(1.42, 1.77)** | **< 0.001** |
| Female (ref = male) | [OR] | [LL, UL] | [p] |
| Age (years) | [OR] | [LL, UL] | [p] |
| Education (ordinal) | [OR] | [LL, UL] | [p] |
| Personal income (ordinal band) | [OR] | [LL, UL] | [p] |
| Urban residence (ref = rural) | [OR] | [LL, UL] | [p] |
| Presence of minor children (ref = none) | [OR] | [LL, UL] | [p] |
| Traditional gender attitudes (1–4) | [OR] | [LL, UL] | [p] |
| Constant | [OR] | [LL, UL] | [p] |
| Observations (n) | 1,520 | — | — |
| McFadden R² | 0.076 | — | — |
| Likelihood-ratio test | [χ²(df)] | — | [p] |

*Note.* For the focal predictor, the standardized association is OR = 1.92, 95% CI (1.64, 2.24) per one-SD increase in the index, with an average marginal effect of +7.4 percentage points per index point. The gender × index interaction was not statistically significant [OR = 0.81, 95% CI (0.65, 1.01), p = 0.066]; index associations by group were OR = 1.44, 95% CI (1.23, 1.68) among women and OR = 1.73, 95% CI (1.48, 2.03) among men.

**Supplementary Table S2 |** Binary logistic regression predicting endorsement of the husband or breadwinner as head of household.

| **Predictor** | **OR** | **95% CI** | **p** |
| --- | --- | --- | --- |
| **Domestic-labor index (per index point)** | **1.27** | **(1.18, 1.37)** | **< 0.001** |
| Female (ref = male) | [OR] | [LL, UL] | [p] |
| Age (years) | [OR] | [LL, UL] | [p] |
| Education (ordinal) | [OR] | [LL, UL] | [p] |
| Personal income (ordinal band) | [OR] | [LL, UL] | [p] |
| Urban residence (ref = rural) | [OR] | [LL, UL] | [p] |
| Presence of minor children (ref = none) | [OR] | [LL, UL] | [p] |
| Traditional gender attitudes (1–4) | [OR] | [LL, UL] | [p] |
| Constant | [OR] | [LL, UL] | [p] |
| Observations (n) | 1,523 | — | — |
| McFadden R² | 0.057 | — | — |
| Likelihood-ratio test | [χ²(df)] | — | [p] |

*Note.* Focal standardized association OR = 1.40, 95% CI (1.26, 1.57) per one-SD increase; average marginal effect +5.1 percentage points. The gender × index interaction was statistically significant [OR = 0.74, 95% CI (0.63, 0.86), p < 0.001]: the association was stronger among men [OR = 1.52, 95% CI (1.34, 1.71)] than among women [OR = 1.12, 95% CI (1.01, 1.23)].

**Supplementary Table S3 |** Binary logistic regression predicting difficulty reconciling paid work and family.

| **Predictor** | **OR** | **95% CI** | **p** |
| --- | --- | --- | --- |
| **Domestic-labor index (per index point)** | **1.17** | **(1.02, 1.34)** | **0.020** |
| Female (ref = male) | [OR] | [LL, UL] | [p] |
| Age (years) | [OR] | [LL, UL] | [p] |
| Education (ordinal) | [OR] | [LL, UL] | [p] |
| Personal income (ordinal band) | [OR] | [LL, UL] | [p] |
| Urban residence (ref = rural) | [OR] | [LL, UL] | [p] |
| Presence of minor children (ref = none) | [OR] | [LL, UL] | [p] |
| Traditional gender attitudes (1–4) | [OR] | [LL, UL] | [p] |
| Constant | [OR] | [LL, UL] | [p] |
| Observations (n) | 1,460 | — | — |
| McFadden R² | 0.024 | — | — |
| Likelihood-ratio test | [χ²(df)] | — | [p] |

*Note.* Focal standardized association OR = 1.26, 95% CI (1.04, 1.52) per one-SD increase; average marginal effect +1.4 percentage points.

**Supplementary Table S4 |** Binary logistic regression predicting naming domestic labor as a barrier to women’s participation.

| **Predictor** | **OR** | **95% CI** | **p** |
| --- | --- | --- | --- |
| **Domestic-labor index (per index point)** | **1.05** | **(0.97, 1.14)** | **0.224** |
| Female (ref = male) | [OR] | [LL, UL] | [p] |
| Age (years) | [OR] | [LL, UL] | [p] |
| Education (ordinal) | [OR] | [LL, UL] | [p] |
| Personal income (ordinal band) | [OR] | [LL, UL] | [p] |
| Urban residence (ref = rural) | [OR] | [LL, UL] | [p] |
| Presence of minor children (ref = none) | [OR] | [LL, UL] | [p] |
| Traditional gender attitudes (1–4) | [OR] | [LL, UL] | [p] |
| Constant | [OR] | [LL, UL] | [p] |
| Observations (n) | 1,485 | — | — |
| McFadden R² | 0.003 | — | — |
| Likelihood-ratio test | [χ²(df)] | — | [p] |

*Note.* The focal association was not statistically significant after adjustment [OR = 1.05, 95% CI (0.97, 1.14), p = 0.224].

**Supplementary Table S5 |** Binary logistic regression predicting marriage-oriented (vs. career-oriented) expectations for daughters.

| **Predictor** | **OR** | **95% CI** | **p** |
| --- | --- | --- | --- |
| **Domestic-labor index (per index point)** | **1.14** | **(1.06, 1.23)** | **< 0.001** |
| Female (ref = male) | [OR] | [LL, UL] | [p] |
| Age (years) | [OR] | [LL, UL] | [p] |
| Education (ordinal) | [OR] | [LL, UL] | [p] |
| Personal income (ordinal band) | [OR] | [LL, UL] | [p] |
| Urban residence (ref = rural) | [OR] | [LL, UL] | [p] |
| Presence of minor children (ref = none) | [OR] | [LL, UL] | [p] |
| Traditional gender attitudes (1–4) | [OR] | [LL, UL] | [p] |
| Constant | [OR] | [LL, UL] | [p] |
| Observations (n) | 1,477 | — | — |
| McFadden R² | 0.014 | — | — |
| Likelihood-ratio test | [χ²(df)] | — | [p] |

*Note.* Focal standardized association OR = 1.21, 95% CI (1.08, 1.34) per one-SD increase; average marginal effect +3.2 percentage points. Traditional gender attitudes were not statistically significant in this model.
